# Human LDL Structural Diversity Studied by IR Spectroscopy

**DOI:** 10.1371/journal.pone.0092426

**Published:** 2014-03-18

**Authors:** José A. Fernández-Higuero, Ana M. Salvador, Cesar Martín, José Carlos G. Milicua, José L. R. Arrondo

**Affiliations:** 1 Unidad de Biofísica (CSIC, UPV/EHU) and Departamento de Bioquímica y Biología Molecular, Universidad del País Vasco, Bilbao, Spain; 2 Departamento de Bioquímica y Biología Molecular, Universidad del País Vasco, Bilbao, Spain; Griffith University, Australia

## Abstract

Lipoproteins are responsible for cholesterol traffic in humans. Low density lipoprotein (LDL) delivers cholesterol from liver to peripheral tissues. A misleading delivery can lead to the formation of atherosclerotic plaques. LDL has a single protein, apoB-100, that binds to a specific receptor. It is known that the failure associated with a deficient protein-receptor binding leads to plaque formation. ApoB-100 is a large single lipid-associated polypeptide difficulting the study of its structure. IR spectroscopy is a technique suitable to follow the different conformational changes produced in apoB-100 because it is not affected by the size of the protein or the turbidity of the sample. We have analyzed LDL spectra of different individuals and shown that, even if there are not big structural changes, a different pattern in the intensity of the band located around 1617 cm^−1^ related with strands embedded in the lipid monolayer, can be associated with a different conformational rearrangement that could affect to a protein interacting region with the receptor.

## Introduction

Low-density lipoprotein (LDL) particles are specialized lipid transport vehicles in the blood. They are the main cholesterol carriers in human plasma, delivering lipids to cells via LDL receptor mediated pathway [1]. A complete maturation of LDL particles is achieved by the action of various lipolytic enzymes and lipid transfer proteins that occurs as a sequential metabolic continuum in the blood [2], [3] Therefore, LDLs represent a heterogeneous group of particles with a density between 1.019 and 1.063 g/ml and an average particle diameter of about 22 nm. These particles are structurally organized into two major compartments: the lipoprotein surface where proteins, phospholipids and free cholesterol are localized; and the lipoprotein core that contains triglycerides and cholesteryl esters. A single copy of apolipoprotein B-100 (apoB-100) surrounds the LDL surface [4] with some regions rich in β-type structures embedded in the lipid domain of the particle [5]. ApoB-100 is a very large monomeric protein formed by 4,536 aminoacids [6]. Lipid composition and apoB-100 conformation determine the structure and physical properties of the LDL particles. It is accepted that a cluster of positively charged residues on apoB-100 (residues 3,359–3,369) interacts with the LDL receptors and the negatively charged glycosaminoglycans of the arterial wall. It has been suggested that apoB-100 is composed of globular domains connected by flexible chains surrounding the LDL and thus stabilizes the structure of the protein–lipid complex [4], [7]. This conformation is likely responsible for the LDL particle structural integrity maintenance. Moreover, the conformational flexibility of these chains most probably controls the structural and compositional changes taking place in the LDL particles[8]. To date, a successful three-dimensional structure analysis of apoB-100 has not been elucidated due to the aforementioned intrinsic conformational flexibility and variability of apoB-100. The available data support the concept that apoB-100 is composed by five distinct alternating α-helical and β-sheet domains: NH- βα1-β1-α2-β2-α3-COOH [9]–[13]. The first 1000 N-terminal residues of apoB-100 and lamprey lipovitellin (residues 1-1074) are highly similar showing 20.1% identity and 39.6% similarity. The atomic crystal structure of this domain is known and harbors important implications for mechanisms involved in the assembly of apoB-containing lipoprotein particles [14], [15]. It has also been recently suggested that the β-strand domains are the major lipid-associating motif in apoB whereas the α-helical domains represent reversible lipid affinity leading to lipid desorb from and reabsorb onto the particle surface [16], [17]. The conformational flexibility between the α-helical and β-strand domains most probably allow the accommodation of apoB-100 to lipoprotein particle changing size. The different apolipoprotein conformations obtained by cryoelectron microscopy images [18] and the epitope exposition dependent on particle size [19] supported the implication of apoB-100 conformation in the interaction with LDL receptor [20]. Moreover, the particle size has been established as a cardiovascular risk factor independent from other dyslipidemias as hypercholesterolemia or hypertriglyceridemia [21]–[23].

It is generally assumed that all the pathological situations, in which the affinity of apoB-100 for cellular receptors is altered, including mutations in the apoprotein and LDL receptor, increase the incidence of atherosclerosis [24]–[26]. The function of apoB-100 is closely related to its structure. Therefore, the characterization of apoB-100 conformational diversity could provide a way to distinguish more atherogenic LDL particles, improving our understanding of its physiological functions as well as the structure-function relationship. To this end, IR spectroscopy could constitute a useful tool because it allows to quickly and easily estimate the secondary structure composition against high-resolution techniques as X-ray diffraction, NMR or cryoEM. Moreover, the infrared spectroscopy also allows determining the lipid/protein ratio of the LDL particles, providing information related to the particle size.

In the present work, we have analyzed different human sera with the aim of finding out characteristic differences among infrared spectra profiles that allow classifying human LDL despite the inter-individual variability.

## Experimental Procedures

### Isolation of LDL

This study was approved by the Research Ethic committee from the University of the Basque Country (Comité de Ética en la investigación y la práctica docente de la Universidad del País Vasco/Euskal Herriko Unibertsitatea; CEID/IIEB). CEID/IIEB ethics committee waived the need for written informed consent because the samples were fully anonymized. Low-density lipoproteins were isolated from blood samples collected from sample donors obtained on different days using a two-step centrifugation according to published procedure [27]. Briefly, blood was collected in EDTA tubes and serum was obtained by centrifugation for 30 min at 12,000 × *g* at 4°C. Blood serum LDL (1.019–1.063 g/ml) was isolated using isopycnic ultracentrifugation. The density of serum samples was adjusted to 1.21 g/ml by the addition of potassium bromide, and then PBS buffer was added, resulting in two phases. The sample was centrifuged at 244,500 × *g* for 19 h 30 min at 4°C. The band corresponding to LDL was recovered and stored at 4°C.

### Analytical methods

The protein content of LDL was determined according to Lowry protein assay [28] with bovine serum albumin (1 mg/ml) as a standard. Serum total cholesterol was measured enzymatically using the reagent kit supplied by BioSystem (BioSystem S.A., Barcelona, Spain) [29].

### Conditioning of the samples for IR spectroscopy analysis

LDL samples were concentrated to approximately 10 mg/ml using Microcon centrifugal filters (Millipore) and dialyzed in a Novagen TM Dialyzer D-Mini Tubes (Merck, Darmstadt, Germany) to exchange the sample buffer by deuterated buffer. The dialyses were carried out at 4°C in a closed container in order to prevent rehydration. The H_2_O-D_2_O exchange was followed by the analysis of infrared spectrum of withdrawn buffer in the same conditions as samples were measured later. The D_2_O buffer was restored for several times until no change was detected as a result of being in contact with the samples. There had been 19 analyzed samples in the study. Each sample was measured by triplicate and in different days to corroborate that there were no errors caused by the methodology. From the 19 analyzed samples, 4 were assigned to A subtype, 9 to B subtype and 6 to C subtype (see Results section and Table S1). To avoid an incorrect interpretation of such frequencies as the ones naturally occurring in population, seven samples were arbitrarily excluded from Table 1 (see Results section) thus showing equal number of each subtype.

**Table 1:** Characterization of LDL samples.

# Sample	Lipid/Protein area %	Height % at 1617 cm^−1^	FWHH^a^ of 1617 cm^−1^ band	Area % of 1617 cm^−1^ band	LDL type
1	34	73.3	15.4	16.5	A
2	34	74.2	15.2	16.9	
3	37	76.8	15.3	17.8	
4	37	82.5	15.5	19.2	
5	41	86.4	15.5	20.3	B
6	47	90.7	15.1	21.5	
7	43	91.0	15.5	21.9	
8	45	91.5	15.3	21.5	
9	46	93.3	14.8	22.6	C
10	43	93.3	14.8	22.8	
11	46	93.4	14.8	22.8	
12	41	93.7	14.7	23.7	

Representative LDL samples are represented by increasing 1617 cm^−1^ absorption percentage of amide I band (considered as 1700–1600 cm^−1^). All the measurements have been performed at least in triplicate with a ±S.D. smaller than 5%. Statistical significance; Lipid/Protein area %: p<0.01 B and C compared to A, not significant C compared to B. Height % at 1617 cm^−1^: p<0.01 B and C compared to A, p<0.01 C compared to B. FWHH of 1617 cm^−1^ band: B compared to A not significant, p<0.01 C compared to A; p<0.01 C compared to B. Area % 1617 cm^−1^ band: p<0.01 B and C compared to A, p<0.01 C compared to B.

Equal number of each subtype is shown to avoid an incorrect interpretation of such frequencies as the ones naturally occurring in population The total number of samples analysed in the study has been 19 and the complete data is shown in Table S1.

a Full Width at Half Height (FWHH).

### Infrared Measurements

Infrared spectra of samples were recorded at 37°C in a Nicolet Nexus 5700 spectrometer equipped with a MCT detector using a Peltier cell (TempCon, Bio Tools) with 25 μm carved calcium fluoride windows. Typically, 370 interferograms were collected per spectrum and then referred to a background, the spectra being obtained with a nominal resolution of 2 cm^−1^.

### Spectral analysis

Quantitative information on the amide I was obtained as previously described [30], [31]. The spectra were digitally subtracted using a spectrum of the last dialysis buffer as a reference for each sample. With the aim of minimize the differences in protein concentration among recorded samples, all spectra were normalized to amide I band area. We have used a narrowing process that preserves the assignment of components [31]. These difference spectra were analyzed by Fourier deconvolution (bandwidth = 18 and k = 2) and Fourier derivation (power = 3 and breakpoint = 0.3) in order to define the number and position of constituent bands of the amide I band. The baselines of normalized spectra were removed before starting the fitting procedure. A two steps fitting method was used as previously described [32]. This procedure consisted in a first iterative stage in which the positions of the component bands obtained by band narrowing techniques were fixed, allowing the estimation of their final widths and heights, and a second stage where band positions were left to change. Gaussian band shape was considered for all component bands [33]. To enhance the accuracy of the comparison of amide I band decompositions, the fittings were performed using, as initial positions, the average wavenumber of each component band obtained from all LDL samples.

### LDL density determination by electric conductivity

After the ultracentrifugation used to isolate LDL from sera, the density gradients were fractionated with a peristaltic pump and introduced in a flow cell equipped with electric conductivity, temperature and ultraviolet (UV) absorbance detectors (ÄKTA, GE Healthcare). Fraction analyses were performed with the Unicorn 4.11 program (Amersham Biosciences). The electric conductivity was used to calculate the density the fractions as previously described [34].

### Statistical analysis

All measurements were performed at least 3 times and levels of significance were determined by a two-tailed Student’s t-test.

## Results

### IR spectrum of human LDL as an indicator of interindividual varibility

**Figure 1:**
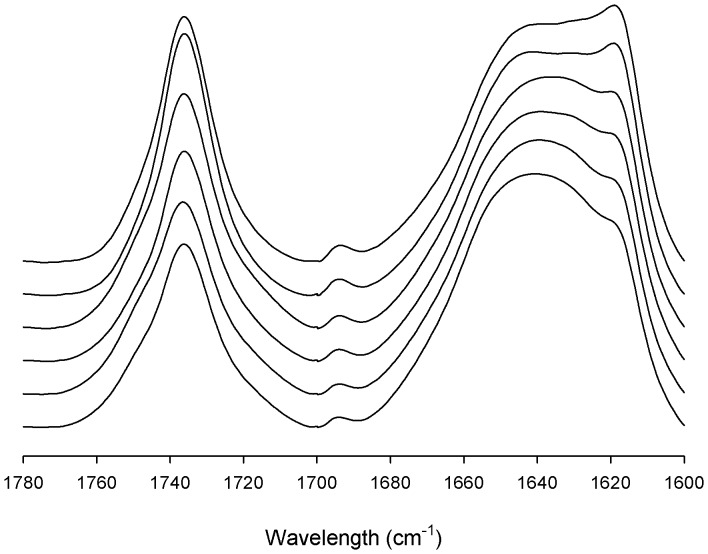
Spectra of lipid esters and amide I band of several LDL samples analyzed. Infrared spectra from 1780 to 1600^−1^ of different LDL samples recorded in D_2_O buffer at 37°C. The spectra are sorted by increasing intensity around 1617 cm^−1^ position, from bottom to top. Two spectra are represented for each class.

LDL infrared spectrum is characterized by two strong absorption bands due to carbonyl group stretching representing the particle components: lipid esters and the amide group of apoB-100 arising mainly of the peptide carbonyl group vibration. The apoprotein amide I band is located from 1700 to 1600 cm^−1^, while the lipid ester absorption appears between 1780 and 1700 cm^−1^ with a maximum around 1736 cm^−1^ that principally corresponds to cholesteryl esters [35]. The amount of esterified lipids contained in VLDL, LDL and HDL particles determine their different densities, which are represented by different contribution of lipid esters and apoproteins in their infrared spectra [36]. Thereby, the ratio between lipid and protein band areas can be used as an indicative of the LDL lipid content, which can distinguish even different density subfractions obtained from the same serum [34]. The LDLs analyzed in this study showed different L/P ratios that seemed to correlate with changes in the amide I band contour (Table 1 and Figure 1). Particularly, the absorption intensity around 1617 cm^−1^ is the more striking variation in the spectra, being clearly lower for denser LDLs. The absorbance at this characteristic position of apoB-100 amide I band is unusual in non aggregated proteins and has been associated with β-strands embedded in the lipid monolayer [33]. These structures have been proposed to vary depending on LDL diameter [37], probably because of the adaptation of the apoprotein to the particle curvature. As previously described [37], we found that LDL with a lower lipid ratio had a reduced β-strands content (Table 1). However, several LDLs with clearly different absorbance at 1617 cm^−1^ had similar L/P ratio, indicating the existence of an apoB-100 structural diversity, not exclusively related to particle density (Table 1). Therefore, interindividual variability in LDL structure and density could be analyzed by IR spectroscopy since each sample presented a characteristic infrared spectrum.

### Spectroscopical classification of human LDLs

As mentioned above, comparison of LDL spectra from different individuals showed a significant diversity concerning the L/P ratio and amide I band shape. Samples with L/P ratio lower than 0.4 presented a low absorbance around 1617 cm^−1^, characteristic of LDLs with small β-strand content. However, the area of 1617 cm^−1^ band was not fully related with the lipid content for lipoproteins with L/P ratio higher than 0.4 (Table 1), suggesting that LDLs with similar density could present different composition in secondary structures. With the aim of classify amide I profiles, the spectra were ordered depending on the absorption at 1617 cm^−1^ expressed as height percentage of the amide I band (Table 1). As was expected the samples with elevated absorbance at this position showed higher contribution of 1617 cm^−1^ band, indicating that these parameters are directly related. The amide I maximum position also depended on the 1617 cm^−1^ band area, between 1645 and 1630 cm^−1^ or around 1620 cm^−1^ for LDL with β-strands content below or above the 22%, respectively. Additionally, the LDLs with higher β-strands content showed a smaller FWHH (Full Width at Half Height) of 1617 cm^−1^ component band, suggesting a different structural environment (Table 1). Based in the parameters described above, three different spectroscopic profiles of LDL can be defined: A-type, characterized by a lipid-protein area ratio below 0.4 and β-strand content lower than 20%; B-type, with L/P ratio above 0.4 and higher β-strand content; and C-type, which can be distinguished from the previous one because of position of the amide I maximum, approximately at 1620 cm^−1^.

### Secondary structure composition of the defined LDLs

Considering the component band positions obtained from Fourier derivation and deconvolution, all LDL spectra were individually band-fitted. The fitting parameters, such as band position and percentage area of each spectral component are shown in Table S2. The assignment of the decomposed bands to specific secondary structure has been carried out as previously described [33]. Briefly, in deuterated media, the α-helix typically absorbs around 1656 cm^−1^, while β-sheets appear close to 1630 cm^−1^. The bands positioned around 1670 and 1680 cm^−1^ are assigned to β-turns. Random or unordered structures appear around 1643 cm^−1^. Finally, the apoB-100 characteristic band at 1617 cm^−1^ is associated to β-strands embedded into LDL monolayer [5], being the 1694 cm^−1^ band its high frequency component [33].

For all analyzed LDL, and similarly to the previously described [33], [36], [37], β-structures are the principal conformation present in the apolipoprotein, divided in β-sheets (25–28%), β-strands (18–24%) and β-turns (8–11%). The random structures are around 22%, and finally, the contribution of α-helix ranges from 19 to 21%. The average composition in secondary structures of each type of LDL is shown in Table 2. The main structural change of apoB-100 among the defined LDL types is the contribution of β-strands, with a difference between A and C types of approximately a 6%. In fact, B-LDL and C-LDL only differ in β sheet and strands contribution, suggesting an exchange between both types of structures. In the case of A-type, the percentage of β-sheets was similar to that of B-type and, the reduction of β-strand contribution could be related with the small increase of other structures (1% of random structures and 2% of α-helix and β-turns; Table 2).

**Table 2:** Secondary Structure contents of LDL types.

Structure	A-LDL	B-LDL	C-LDL
β -Turn	10.8±0.6	8.9±0.5	8.7±0.5
α – helix	21.3±0.6	19.4±0.4	19.7±0.4
Random	22.7±0.7	21.8±0.6	21.7±0.3
β Sheet	27.0±0.6	28.0±0.7	25.7±0.3
β Strand	18.3±1.2	21.8±0.7	24.3±0.8

Values shown represent the mean ± standard deviation, with a sample size of 4 (A-LDL), 9 (B-LDL) and 6 (C-LDL), respectively. The data from all the samples analyzed in this study shown in Table S2 were considered to calculate both the mean and standar deviation.

**Figure 2:**
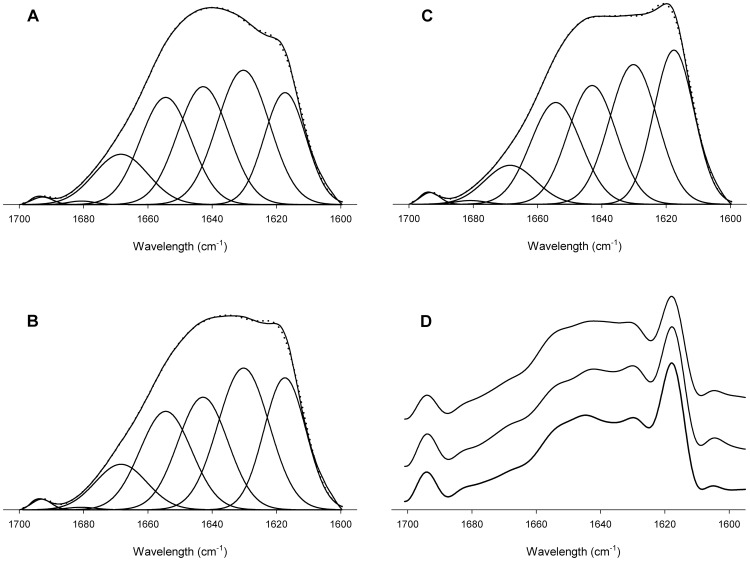
Amide I band representative decomposition of LDL-types. The spectra were obtained in D_2_O buffer at 37°C. A) LDL A-type (corresponding to sample 3 shown in Table 1); B) LDL B-type (corresponding to sample 8 shown in Table 1); C) LDL C-type (corresponding to sample 12 shown in Table 1) and D) deconvolved spectra of samples 3, 8 and 12 (spectra are offset from top to the bottom). The parameters corresponding to the component bands are reflected in Table S2. The spectra shown in the figure were chosen because they are the closest ones to the mean values shown in Table 2 for the 4 samples of LDL-A subtype, 9 samples of LDL-B subtype and 6 samples of LDL-C subtype and therefore can be considered as representative of each subtype.

Even with the clear differences between the profiles of the amide I band of the LDL types (Figures 1 and 2), the quantitative estimation of their secondary structure composition did not show big percentage differences. The changes in the area of a band are related not only with the amount of structure present but also with variations in height and width, arising the latter from the librational vibration associated with the freedom of the molecules, implying changes in the environment of the protein. Because of the huge size of apoB-100 protein (4,536 aminoacids, 512 kD), small area changes in a band component may also involve a significant number of aminoacids that are being rearranged in a different tridimensional environment. In fact, a 3% difference would imply more than 100 aminoacids. Therefore, a small variation in average secondary structure composition of apoB-100 may suppose a considerable structural rearrangement of the protein in the particle.

## Discussion

It is clearly established the relationship between the high LDL level in blood and coronary heart disease (CHD). Total cholesterol determination is a usual way to evaluate the risk of having cardiovascular accident because LDL is the main cholesterol carrier[38]. However, this is not an accurate diagnosis due to the presence in plasma of other non atherogenic particles, such as very low density lipoprotein (VLDL) and high density lipoprotein (HDL). The diagnosis of CHD in patients without increased LDL pointed out the question if other factors such as LDL profile could lead to disease [39]–[41]. Size and density of LDL vary among individuals, constituting a considerable heterogeneous group of particles [42]. Particularly, a lipoprotein profile characterized by large proportion of small and dense LDL (sdLDL) is related to elevated incidence of atherosclerosis [22], [23], [43], [44] probably because of its higher susceptibility against oxidation [45], [46] and higher affinity for proteoglycans [47], that facilitates its accumulation into vascular wall. Moreover, these sdLDL seem to have less affinity for its receptor implying a fall in hepatic absorption and an increased time of residence in plasma [48], [49]. It has been demonstrated a significant association between sdLDL with a threefold increased risk of myocardial infarction. This could be related to the fact that in patients presenting a sdLDL phenotype, plasma levels of HDL are decreased whereas VLDL and triglyceride levels are increased [50]. These harmful characteristics of smaller and denser particles, that modify its functional activity, can be related to apoB-100 conformation as changes in the exposition of several epitopes due to size reduction and the modification of the lipid content and proteins occurring during the intravascular transformation from VLDL to LDL which have been detected using monoclonal antibodies [19].

The most widely used methods for determining LDL subfraction phenotype provide information about LDL size or density distribution but they are not suitable to obtain information related to apoB-100 structure. Among them are gradient gel electrophoresis (GGE) [51], proton NMR spectroscopy [52], [53], analytical ultracentrifugation [54] and ion mobility [55]. Although it has been reported that they are not completely accurate [56], very recently it has been described an analytical agreement among results obtained by these methods by different laboratories [57]. According to that study, phenotype of the LDL subfractions can be classified as pattern A (larger, less dense particles), pattern B (smaller, more dense particles), and an intermediate pattern (A/B).

**Figure 3:**
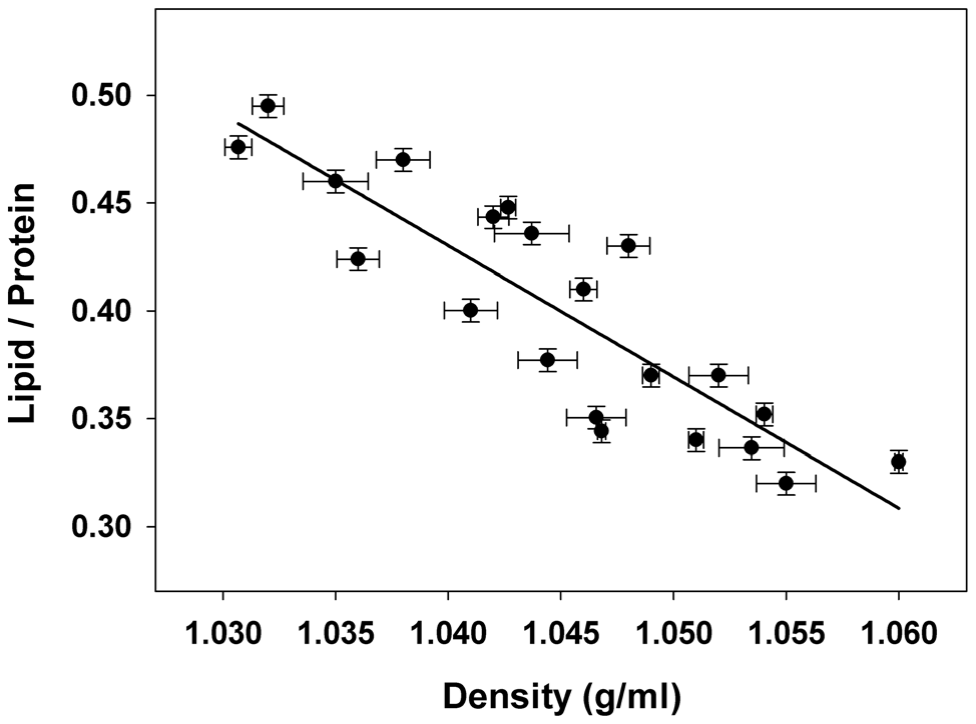
Correlation between density and lipid/protein area ratio of different LDL samples. LDL density determination by electric conductivity was measured as described in Methods. The lipid/protein area ratios were calculated from the normalized spectra after baseline subtraction, integrating the area from 1770 to 1700 cm^−1^ for the lipid ester band and between 1700 and 1600 cm^−1^ for protein. Each sample was measured at least in triplicate. The correlation coefficient obtained was r^2^ = 0.7678.

Our IR spectroscopic study demonstrates a clear structural diversity among individuals analyzed, either in lipid content as well as in the secondary structure of apoB-100. Moreover, the lipid relative content of each LDL measured by the area ratio between lipid esters band and the amide I band gives a comparative idea of the size and density of the particle as previously reported for LDL subfractions [37]. In the present work, we have also confirmed the correlation between density and lipid/protein area ratio of different LDL samples (Figure 3). Thus, three types of LDL have been proposed using four different spectroscopic parameters: L/P ratio, intensity at 1617 cm^−1^, band area at 1617 cm^−1^ and FWHH. The different LDL spectra profiles could be related to LDL phenotypes recently described since pattern B, which is characterized by smaller and more denser particles, could be ascribed to A-type, whereas larger LDLs of pattern A and pattern (A/B) could be associated with B- and C-type here proposed [57]. The results obtained by IR spectroscopy not only provide information related to size and density of LDL, but also information about the secondary structure composition of the analyzed LDL particles. Therefore IR spectroscopy is an adequate methodology to characterize LDL phenotype because the obtained additional information regarding apoB-100 structure complements the results obtained by other methodologies. In addition, the conformational information of apoB-100 provided by IR spectroscopy could be associated to different atherogenicity of LDLs. Accordingly to a previous work, denser LDLs present a minor content of β-strands [37], but other particles with similar L/P ratio show a different amide I band profile, indicating differences in the secondary structure composition. Noteworthy is that a correct apoB-100 structure is needed to maintain the integrity of LDL and LDL-cholesterol levels in plasma. It has been well documented that the apoB-100 highly conserved receptor-binding site is stabilized by the interaction of Arg3500 with Trp4369, since a single point amino acid mutation (Arg3500 to Glu3500), which is outside this region, completely abolishes receptor recognition. This effect shows that a single aminoacid alteration could destabilize important clusters for the apoB-100 conformation thus altering the three-dimensional protein structure. Therefore, both the diameter of the LDL particle and apoB-100 conformation have important consequences for a correct binding of apoB-100 to the LDL receptors [58]. So, the structural characterization of apoB-100 would be a useful tool for determining the potential atherogenicity of LDLs.

It would be interesting to analyze lipoproteins from physiologically tested samples in order to find out a relationship between the atherogenicity and IR spectrum of the lipoprotein. Therefore, it is important to analyse the IR spectrum of LDL particles with different mutations in apoB-100 in order to identify any spectrum pattern in the mutant LDLs that could be useful for an accurate prediction of CHD risk.

## Conclusion

LDL IR spectroscopic analysis constitutes a tool for consistent LDL phenotype classification, which could help in the diagnosis of CHD. Changes in the organization of the apoB-100 protein, possibly related to possible atherogenic properties, can be monitored by changes in the β-strands band intensity and width, offering a simple way of classifying LDL particles. IR also offers the possibility to evaluate the effect of apoB-100 mutations in the protein structure that could lead to a loss of receptor binding causing hypercholesterolemia.

## Supporting Information

Table S1 Characterization of LDL samples.
(DOC)

Table S2 Amide I band decomposition of analyzed LDL samples.
(DOC)
