# Supplementary material for: Human LDL Structural Diversity Studied by IR Spectroscopy
Source: PLoS One. 2014 Mar 18;9(3):e92426. doi: 10.1371/journal.pone.0092426 (PMC3958539; doi:10.1371/journal.pone.0092426)
Supplement: Table S1 — Characterization of LDL samples. (DOC) [file pone.0092426.s001.doc]

**Table S1 – Characterization of LDL samples.**

| **# Sample** | **Lipid / Protein**  **area %** | **Height %**  **at 1617 cm^-1^** | **FWHH^a^ of**  **1617 cm^-1^ band** | **Area % of**  **1617 cm^-1^ band** | **LDL**  **type** |
| --- | --- | --- | --- | --- | --- |
| 1 | 34 | 73.3 | 15.4 | 16.5 | **A** |
| 2 | 34 | 74.2 | 15.2 | 16.9 |
| 3 | 37 | 76.8 | 15.3 | 17.8 |
| 4 | 37 | 82.5 | 15.5 | 19.2 |
|  | 43 | 85.6 | 15.1 | 20.2 | **B** |
| 5 | 41 | 86.4 | 15.5 | 20.3 |
|  | 44 | 86.8 | 15.2 | 20.6 |
|  | 41 | 88.9 | 15.3 | 21.1 |
|  | 44 | 90.1 | 15.1 | 21.6 |
| 6 | 47 | 90.7 | 15.1 | 21.5 |
|  | 47 | 90.7 | 15.3 | 21.1 |
| 7 | 43 | 91.0 | 15.5 | 21.9 |
| 8 | 45 | 91.5 | 15.3 | 21.5 |
| 9 | 46 | 93.3 | 14.8 | 22.6 | **C** |
| 10 | 43 | 93.3 | 14.8 | 22.8 |
| 11 | 46 | 93.4 | 14.8 | 22.8 |
|  | 43 | 93.5 | 14.8 | 23.8 |
| 12 | 41 | 93.7 | 14.7 | 23.7 |
|  | 53 | 94.2 | 15.0 | 24.1 |

LDL samples are represented by increasing 1617cm^-1^ absorption percentage of amide I band (considered as 1700-1600 cm^-1^). All the measurements have been performed at least in triplicate with a ±S.D. smaller than 5 %. Statistical significance; Lipid/Protein area %: p<0.01 B and C compared to A, not significant C compared to B. Height % at 1617 cm^-1^: p<0.01 B and C compared to A, p<0.01 C compared to B. FWHH of 1617cm^-1^ band: B compared to A not significant, p<0.01 C compared to A; p< 0.01 C compared to B. Area % 1617cm^-1^ band: p<0.01 B and C compared to A, p<0.01 C compared to B. From the 19 analyzed samples, 4 were assigned to A subtype, 9 to B subtype and 6 to C subtype (see Results section). To avoid an incorrect interpretation of such frequencies as the ones naturally occurring in population, seven samples (listed in red) were arbitrarily excluded to show equal number of each LDL subtype in Table 1.

^a^ Full Width at Half Height (FWHH)
