# Supplementary material for: Human LDL Structural Diversity Studied by IR Spectroscopy
Source: PLoS One. 2014 Mar 18;9(3):e92426. doi: 10.1371/journal.pone.0092426 (PMC3958539; doi:10.1371/journal.pone.0092426)
Supplement: Table S2 — Amide I band decomposition of analyzed LDL samples. (DOC) [file pone.0092426.s002.doc]

**Table S2 – Amide I band decomposition of analyzed LDL samples**

|  | **Component band position (cm^-1^)** | | | | | | | **LDL**  **type** |
| --- | --- | --- | --- | --- | --- | --- | --- | --- |
| **# Sample** | **1617** | **1630** | **1643** | **1654** | **1669** | **1680** | **1694** |  |
| **1** | 16.5 | 26.7 | 23.0 | 21.8 | 10.8 | 0.5 | 0.7 | **A** |
| **2** | 16.9 | 26.5 | 23.0 | 21.7 | 10.8 | 0.5 | 0.6 |
| **3** | 17.8 | 26.9 | 23.1 | 20.8 | 10.4 | 0.4 | 0.6 |
| **4** | 19.2 | 27.8 | 21.6 | 20.7 | 9.8 | 0.2 | 0.6 |
|  | 20.2 | 27.2 | 22.6 | 19.8 | 9.2 | 0.2 | 0.7 | **B** |
| **5** | 20.3 | 29.0 | 21.0 | 20.0 | 8.9 | 0.1 | 0.7 |
|  | 20.6 | 27.6 | 22.3 | 19.7 | 9.0 | 0.1 | 0.6 |
|  | 21.1 | 27.8 | 21.5 | 19.4 | 9.0 | 0.3 | 0.8 |
|  | 21.6 | 27.6 | 21.9 | 19.3 | 8.7 | 0.1 | 0.7 |
| **6** | 21.5 | 27.9 | 22.2 | 18.9 | 8.7 | 0.1 | 0.7 |
|  | 21.1 | 29.1 | 22.0 | 19.1 | 8.0 | 0.0 | 0.7 |
| **7** | 21.9 | 28.1 | 21.0 | 19.5 | 8.3 | 0.5 | 0.8 |
| **8** | 21.5 | 27.6 | 22.2 | 18.9 | 9.1 | 0.0 | 0.8 |
| **9** | 22.6 | 25.7 | 22.1 | 19.9 | 7.8 | 0.8 | 1.0 | **C** |
| **10** | 22.8 | 26.1 | 21.4 | 19.7 | 8.2 | 0.8 | 1.0 |
| **11** | 22.8 | 25.9 | 21.9 | 20.2 | 7.7 | 0.7 | 0.9 |
|  | 23.8 | 25.1 | 21.5 | 19.4 | 7.9 | 1.1 | 1.2 |
| **12** | 23.7 | 25.8 | 22.0 | 19.7 | 7.6 | 0.4 | 0.9 |
|  | 24.1 | 25.4 | 21.6 | 19.0 | 8.0 | 0.9 | 1.0 |

Values are the percentage of the contribution of each component band considered.

From the 19 analyzed samples, 4 were assigned to A subtype, 9 to B subtype and 6 to C subtype (see Results section). To avoid an incorrect interpretation of such frequencies as the ones naturally occurring in population, seven samples (listed in red) were arbitrarily excluded to show equal number of each LDL subtype in Table 1.
